# Sociodemographic characteristics and healthcare utilization of infants with SARS-CoV-2 in the U.S

**DOI:** 10.1038/s41372-023-01659-8

**Published:** 2023-03-31

**Authors:** Leah Yieh, Ashley Y. Song, Cynthia L. Gong, Kshama Shah, Yijie Li, Ashwini Lakshmanan

**Affiliations:** 1 Fetal and Neonatal Institute, Division of Neonatology, Children’s Hospital Los Angeles, Department of Pediatrics, Keck School of Medicine, University of Southern California, Los Angeles, CA USA; 2 Leonard D. Schaeffer Center for Health Policy and Economics, School of Pharmacy, University of Southern California, Los Angeles, CA USA; 3 Department of Mental Health, John Hopkins Bloomberg School of Public Health, Baltimore, MD USA; 4 Department of Pediatrics, NorthShore University HealthSystem, Evanston, IL USA; 5 Department of Health Systems Science, Bernard J. Tyson Kaiser Permanente School of Medicine, Pasadena, CA USA

**Keywords:** Risk factors, Health services

## Introduction

The severe acute respiratory syndrome coronavirus 2 (SARS-CoV-2) pandemic has exacerbated existing health disparities in the United States [1]. After the onset of the pandemic, hospitals empirically screened patients for SARS-CoV-2. Many children were incidentally found to have SARS-CoV-2 though the primary reason for their encounter may have been unrelated [2]. We aimed to describe sociodemographic characteristics of infants less than one year of age diagnosed with SARS-CoV-2 alongside healthcare utilization while distinguishing between those with confirmed versus incidental infection.

## Methods

We performed a retrospective cohort analysis using the Cerner COVID-19 De-Identified Dataset, which consists of 110 health systems throughout the U.S. with a total of 3.8 million patients. Data includes all patients with a SARS-CoV-2 exposure or infection code and initial and subsequent encounters (inpatient, emergency room, urgent care, or observation) [3]. We included all infants less than one year of age with positive SARS-CoV-2 diagnosis and/or lab test from December 1, 2019 to December 31, 2021. Infants were categorized as either positive SARS-CoV-2 lab test without a SARS-CoV-2 diagnosis (presumed to be incidental finding not related to primary reason for encounter) or positive SARS-CoV-2 lab test with a SARS-CoV-2 diagnosis (presumed to be confirmed SARS-CoV-2 infection as primary reason for seeking healthcare). Multivariable linear and logistic regression models were used to identify factors associated with length of stay (LOS) and multiple encounters, respectively. *E*-values were calculated to account for unmeasured confounders. All analyses were completed using R version 4.1.0. This study was approved by Children’s Hospital Los Angeles Institutional Review Board (CHLA-20-00236).

## Results

A total of 85,008 infants had a positive SARS-CoV-2 lab test, of which 38,396 also had a documented SARS-CoV-2-related diagnosis (54.8% male; 58.3% White, 15.4% Black, 1.2% American Indian, 2.5% Asian/Pacific Islander, 14.2% other; 38.8% Hispanic; 64.3% government insurance). The remaining 46,612 infants with a positive SARS-CoV-2 lab test had no associated SARS-CoV-2 diagnosis (54.8% male; 55.7% White, 17% Black, 1.2% American Indian, 2.4% Asian/Pacific Islander, 12.2% other; 39.2% Hispanic; 65.4% government insurance).

After adjusting for sex, race/ethnicity, payer type, and number of secondary diagnoses and procedures at the initial encounter, Black infants with a confirmed SARS-CoV-2 infection were more likely to have a longer LOS by 0.92 days (95% CI 0.59 to 1.24, *p* < 0.001) versus White infants. For those with an incidental SARS-CoV-2 infection, American Indian infants were at higher risk of increased LOS by 4.39 days (95% CI 2.98 to 5.80, *p* < 0.001), Black infants by 0.83 days (95% CI 0.44 to 1.23, *p* < 0.001), and Mixed race infants by 1.55 days (95% CI 0.44 to 2.66, *p* = 0.006) compared to White infants. Privately-insured infants were likely to have a shorter LOS by 0.84 days (95% CI −1.15 to −0.54, *p* < 0.001) versus government-insured infants.

In the logistic regression model (Table 1), those with a confirmed and incidental SARS-CoV-2 infection who presented again to the same health system (had subsequent encounters) were more likely to be Black versus White and Hispanic versus non-Hispanic. For confirmed and incidental infections, infants with private insurance versus government insurance were less likely to present again.

**Table 1:** Odds Ratios and *E*-values of Factors Associated with Subsequent Encounters.

	Confirmed infection					Incidental infection				
Index encounter variables	OR	95 % CI	*p*-value	*E*-value	CI bound	OR	95% CI	*p*-value	*E*-value	CI bound
Sex										
Male	Ref					Ref				
Female	0.89	0.83, 0.95	<0.001	1.31	1.19	0.91	0.85, 0.96	0.002	1.27	1.17
Race										
White	Ref					Ref				
Black	1.26	1.14, 1.40	<0.001	1.49	1.33	1.39	1.28, 1.52	<0.001	1.64	1.52
Other^a^	0.78	0.71, 0.86	<0.001	1.52	1.37	0.68	0.62, 0.74	<0.001	1.72	1.59
Ethnicity										
Non-Hispanic	Ref					Ref				
Hispanic	1.15	1.06, 1.24	<0.001	1.35	1.20	1.39	1.29, 1.49	<0.001	1.64	1.53
Payer										
Government	Ref					Ref				
Private	0.77	0.71, 0.83	<0.001	1.54	1.43	0.71	0.66, 0.77	<0.001	1.66	1.54
Length of stay	1.00	0.99, 1.00	0.05	1.00	1.00	0.99	0.99, 1.00	0.65	1.08	1.00
Number of diagnoses	1.02	1.01, 1.03	<0.001	1.11	1.08	1.01	1.00, 1.02	0.003	1.08	1.00
Number of procedures	0.99	0.99, 1.00	0.02	1.08	1.00	0.99	0.98, 1.00	0.05	1.08	1.00

^a^Other race includes Native American, Asian/Pacific Islander, Other, Mixed.

To assess the impact of unmeasured confounders, we calculated *E*-values using odds ratios (ORs) from the logistic regression model for subsequent encounters (Table 1). There were relatively low *E*-values for all the variables, even those with significant *p* values.

## Discussion

In our study of infants with confirmed and incidental SARS-CoV-2 infection, we found racial disparities in both groups in terms of LOS and subsequent healthcare encounters. We also found socioeconomic disparities in that government-insured infants were likely to have a longer LOS and subsequent healthcare encounters, which may represent disparities in access to a medical home.

Unlike previously published studies on SARS-CoV-2, we quantified the extent of unmeasured confounding with the use of *E*-values [4]. A relatively low *E*-value, as we found in our study, implies there is weak evidence for an observed effect such that an unmeasured confounder could explain away the observed results. Some unobservable confounders may include social determinants of health, maternal immunization status, limited availability of testing supplies early in the pandemic, and local SARS-CoV-2 transmission rates. Additionally, our dataset only included the beginning of the time period in which the B.1.1.259 (Omicron) variant became dominant in the U.S. After Omicron became predominant, infants and toddlers were hospitalized at five times the rate of the Delta variant peak [5].

These limitations highlight the need for transparency and data sharing in our disjointed healthcare system, the deficits of which have been further exposed during the pandemic. Mandatory reporting to a national pediatric registry with granular data, including social determinants of health, would facilitate more accurate assessments of the impact of SARS-CoV-2 and identify targets for health equity interventions.
